# Missing infrastructure for real-world predictive AI impact

**DOI:** 10.1136/bmjhci-2025-101954

**Published:** 2026-06-19

**Authors:** Benjamin I Perry, Ben Hammond, Dominic Oliver, Ashley Mushambi, Carina Andrews, Niels Peek, Ben Van Calster, Laure Wynants, Annabel E L Walsh, Joseph E Alderman, Shuqing Si, Alastair K Denniston, Simon J Griffin, Joie Ensor

**Affiliations:** 1 Institute for Mental Health, University of Birmingham, Birmingham, UK; 2 Birmingham and Solihull Mental Health NHS Foundation Trust, Birmingham, UK; 3 Applied Health Sciences, University of Birmingham, Birmingham, UK; 4 Department of Psychiatry, University of Oxford, Oxford, UK; 5 Expert by Experience, NA, UK; 6 THIS Institute, University of Cambridge, UK; 7 Department of Development and Regeneration, Katholieke Universiteit Leuven, Leuven, Flanders, Belgium; 8 Care and Public Health Research Institute, Maastricht University, Maastricht, Limburg, Netherlands; 9 The McPin Foundation, London, UK; 10 Inflammation and Ageing, University of Birmingham College of Medical and Dental Sciences, Birmingham, UK; 11 Centre for Regulatory Science & Innovation, Birmingham Health Partners, Birmingham, Birmingham, UK; 12 Public Health and Primary Care, University of Cambridge, Cambridge, UK

**Keywords:** Artificial intelligence, Implementation Science, Data Science, Clinical Governance

## Abstract

Health systems are under increasing pressure as chronic disease becomes the dominant burden of care. Addressing this requires earlier detection and prevention, and predictive artificial intelligence (AI) tools are a demonstrable means of targeting interventions more effectively. Despite extensive research activity and substantial public funding, few predictive AI tools are translated effectively into routine care. We argue that the primary translational barrier is now organisational rather than solely methodological: without defined institutional ownership and long-term stewardship, including to support obtaining and maintaining regulatory certification and navigating governance structures, predictive AI tools cannot progress beyond print. Three interdependent recommendations are proposed to address this problem.

##  The missing infrastructure for real-world predictive AI impact

### Data-driven prevention is key to improving healthcare

Chronic disease now accounts for the majority of healthcare activity and expenditure, placing sustained pressure on the National Health Service (NHS) and other health systems.^1 2^ Enhancing earlier detection and prevention is therefore a core strategic priority.^3^ Recent national initiatives in the UK, including the Life Sciences Vision and recommendations to treat health data as critical national infrastructure, reflect growing momentum for such data to support more proactive and personalised care,^4^ though their downstream impacts may take time to materialise.

Predictive artificial intelligence (AI) tools can be broadly defined as models developed to predict an individual’s risk of current or future health outcomes. While they may be developed using a range of different methodologies including traditional statistical, machine-learning or AI-based methods, they share similar considerations regarding interpretability, validation requirements, regulation and clinical usability. Predictive AI tools present a clear opportunity to bridge the increasing availability of anonymised routine health data with the necessity to shift toward earlier detection and prevention in healthcare. Predictive AI tools that are thoughtfully designed to inform actionable risk-based decisions around diagnosis, prevention, screening and treatment promise a means to target interventions more effectively and improve benefit–harm ratios.^5^

The potential for large-scale impact has driven a rapid expansion in predictive AI research across the past two decades. A recent systematic review and regression modelling extrapolation study estimated that nearly 250 000 articles reporting the development of prediction models were published between 1950 and 2024, representing over 10 000 new models annually.^6^ Further, identifiable funding awards from major UK public funders for predictive AI-related research totalled over £170 m in the last 10 years alone. Against this backdrop of substantial research activity and investment, the extent to which predictive AI tools have delivered measurable improvements in clinical care remains uncertain.

We argue that this translational gap goes beyond well-recognised methodological concerns to a more fundamental problem: the absence of organisational, technical, regulatory and commercial infrastructure to support the safe adoption and long-term maintenance and refinement of predictive AI tools in practice.

## The current landscape of predictive AI: research waste

Most predictive AI tools remain confined to the pages of scientific journals, and prospective research shows a dearth of implementation or impact evidence among the relatively small proportion that are translated into practice.^7^ Systematic reviews across biomedicine coalesce around findings of shortcomings including poor design and fit within existing care pathways, incomplete reporting,^8^ insufficient evaluation,^9^ equity concerns^10^ and inconsistent peer review.^11^ These shortcomings culminate in a state of academic insufficiency with significant clinical and academic opportunity costs, and have prompted concerted efforts to improve conduct and reporting.^12 13^ Early signs suggest that these efforts are beginning to bear fruit.^14^ However, as methodological standards strengthen, other barriers further along the translational pathway are becoming more apparent.

## The transdisciplinary nature of predictive AI translation

Translating a predictive AI tool into clinical practice is inherently transdisciplinary. Yet most predictive AI research activity remains concentrated on the early stages of model development and statistical evaluation. QRISK3 for cardiovascular disease primary prevention and FRAX for osteoporotic fracture risk are among a relatively small number of tools that are embedded in clinical guidelines. These examples illustrate the value of sustained stewardship and integration into routine clinical systems. However, guideline-embedded predictive AI tools remain exceptions. To increase the throughput of tools that can benefit patients, research pipelines must extend beyond model performance to encompass how tools are built, communicated and embedded into care.

This requires coordinated work across multiple domains. Technical and human factors engineering are needed to implement models as reliable, usable software within clinical systems.^15^ Stakeholder engagement and qualitative research are necessary to co-design health-risk communication, user interfaces and care integration plans with clinicians, patients and service leaders.^16^ Behavioural science is required to support effective clinical decision making and behaviour change motivation,^17^ while implementation science helps to design and test strategies for integrating tools into workflows and organisations.^18^ Prospective impact evaluation then examines whether these combined efforts actually change processes, outcomes and equity in practice.^19^

These translational components are not neatly sequential and do not occur in isolation. Decisions about software design influence how clinicians and patients interpret risk estimates; qualitative insights shape implementation strategies; early implementation work reveals unforeseen usability, safety or equity issues that prompt refinement of the model and/or its implementation. Translation is therefore better conceived as an ongoing research–implementation cycle in which evidence, design and practice continuously inform one another, rather than a linear sequence from ‘model development’ to ‘deployment’.

## Predictive AI research and regulation

Predictive AI tools that influence clinical decision-making are increasingly subject to regulatory scrutiny as medical device software, appropriately reflecting the principle that patients should benefit from safe and effective technologies.

A regulated predictive AI tool requires an entity that can assume legal manufacturer status, maintain a quality management system, support postmarket surveillance and ensure ongoing safety and consistency over time. Meeting these responsibilities entails sustained legal, governance, technical and commercial capacity, and an organisational commitment to act as custodian of a live clinical product rather than as host of a time-limited research project. These expectations extend beyond the traditional expertise of most research teams and are rarely recognised or systematically planned for at the point predictive AI projects are conceived.

## The regulatory landscape is shifting

Regulatory frameworks for medical device software are tightening internationally. For example, the Medicines and Healthcare products Regulatory Agency (MHRA) Medical Devices Regulations 2002 (as amended) (UK MDR)^20^ and European Union (EU) Medical Device Directive^21^ previously similarly classified most predictive AI tools as *‘*low risk’. Recent changes to EU medical device regulation (EU Medical Devices Regulation 2021) depart from the UK MDR, now considering any software ‘intended to provide information used to take decisions with diagnostic or therapeutic purposes’ (including diagnosis, prevention, monitoring, prediction, prognosis, treatment or alleviation of disease) to be upclassified to at least the higher-risk Class IIa.^22^ This has clear implications, with compliance requirements, documentation workload, time to market and overall operational burden and financial costs scaling with regulatory risk classification.

Realigning of regulatory frameworks is expected across other global regulators including Great Britain in the coming years.^23^ Increased regulatory scrutiny strengthens patient safety but also increases the organisational and resourcing requirements for deployment, where impacts may disproportionately be felt among smaller institutions or less well-resourced settings. These regulatory developments will shape what is required of any research group or organisation seeking to deploy predictive AI clinically and underscore the importance of sustainable translational arrangements.

## Translational impact in predictive AI from academia is possible

CanRisk, based on the BOADICEA algorithm,^24^ has been refined over two decades of international collaborative research and extensive stakeholder involvement, and has emerged as a strong institutional impact case.^25^ A key step in enabling impact was organisational: in 2020, the University of Cambridge registered as the legal manufacturer of CanRisk with the MHRA. This established the governance, quality management and postmarket arrangements required to allow CanRisk to progress through to clinical use. Now endorsed by the National Institute for Health and Care Excellence (NICE) in the UK alongside similar endorsements internationally, CanRisk has supported over 3 million cancer risk assessments globally, guiding important decisions on cancer screening and prevention.^26^

More recently, in 2025, the University of Birmingham followed a similar path with PsyMetRiC,^27^ a cardiometabolic predictive AI tool for young people with severe mental illness.^28^ Registering with the MHRA as the device manufacturer has enabled regulatory certification, permitting routine clinical use in Great Britain. The University’s Operating Division model is now being leveraged by the research team to support self-sustainability, including postmarket monitoring and iterative refinement as evidence, workflows and populations change. These examples illustrate that it is possible for academic institutions to assume manufacturer responsibilities and build the governance arrangements required for clinical use, but such arrangements are uncommon.

Commercialisation via spin-out or partnership can provide an alternative route to sustainability, and academic institutions are increasingly supportive of such pathways. However, predictive AI tools developed through public or charitable funding are often expected to adhere to open science principles.^12^ These principles are critical for academic, clinical, policy and public confidence in research, yet may simultaneously limit the protection of intellectual property or attractiveness to investors. This can create a paradox where the most methodologically-rigorous, transparent predictive AI tools are the least commercially attractive.

For example, QRISK3 provides an openly available reference implementation supporting scrutiny and reuse, but limited proprietary defensibility means commercial incentives may concentrate on integration, workflow embedding and service models rather than investment in the underlying transparent model itself. Conversely, when tools are developed commercially, disclosure of development, validation and performance details may be constrained by business considerations, limiting independent evaluation even where transparency would increase trust. While tools developed in less open settings may therefore progress more readily, concerns can sometimes remain over transparency and trustworthiness.^29^ Emerging approaches to enable independent evaluation of predictive AI research without requiring commercial disclosure may help address this tension.^30^

## The desperate need for an integrated infrastructure for predictive AI translation

Taken together, the patterns described of extensive research waste, the demands of transdisciplinary translation and escalating regulatory expectations point to a deeper, system-level problem. Predictive AI research is limited by the lack of organisational structures that can both host tools as regulated medical devices and sustain the ongoing research activities required to keep them clinically useful. While many tools are developed within academic–health partnerships, responsibility for moving a model beyond publication is typically diffuse, with no clearly designated entity to coordinate development, regulatory certification, implementation and postmarket oversight.

The transdisciplinary expertise needed for translation exists across research and NHS organisations, but it is often fragmented across departments and directorates without a mechanism to align it. This fragmentation makes it difficult not only to take on manufacturer responsibilities, but also to run the continuous cycle of updating, re-evaluating and reintegrating tools as evidence, workflows and populations change.

Liability and ownership are key bottlenecks. Assuming ‘legal manufacturer’ status concentrates legal and financial responsibility for regulatory compliance, post-market surveillance and incident response, which can deter organisations from acting as long-term custodians. In practice, universities may be able to draw on established technology transfer/commercialisation structures to support governance and risk management, whereas NHS provider organisations may face tighter operational constraints and lower board-level risk tolerance for product-like liabilities.

Institutions are not typically well-positioned to support predictive AI as a living clinical technology rather than as a one-off research output. The challenge is therefore systemic, not scientific: aligning capabilities, assigning ownership and creating pathways through which predictive AI tools can move coherently between research, regulation and routine use.

Establishing mechanisms to bring these capabilities together would enable repeatable, end-to-end translational pathways. While the investment required may seem large for a single tool, the value becomes clear when it supports a pipeline of tools at institutional, regional or national scale.

## A proposal to improve the translational environment for predictive AI research

To address this problem, we propose three system-level actions developed iteratively through multidisciplinary author consensus: obliging clear plans for translation before research is funded; establishing institutional or regional units to support regulatory certification and the research lifecycle; and leveraging national infrastructure to enable implementation, postmarket activities and impact evaluation at scale (box 1). These recommendations inevitably bring challenges, particularly around funding constraints, workforce capacity and the complexity of governance and accountability across organisations. However, the potential rewards eclipse these barriers, not only through academic advancements but through real-world impact at patient and population level.

Box 1 **Three recommendations to improve the translational environment for predictive AI**

**Only fund predictive AI research with stage-appropriate translational considerations**

- Funders should apply greater scrutiny to predictive AI grant proposals’ translation plans before awarding funds, proportionate to project maturity. For early-stage proposals, this should at minimum articulate the intended care pathway and decision impacted, likely deployment setting, initial stakeholder involvement and anticipated regulatory and governance considerations. For later-stage proposals, expectations should be stronger: detailed implementation plans (integration, monitoring, equity and safety and secured translational support), regulatory strategy, ongoing and comprehensive stakeholder engagement, evaluation design and resourcing for deployment. Where appropriate, translational workstreams and funds should be embedded within the initial award, with explicit stop–go criteria at key translational milestones (eg, care pathway fit; equitable predictive performance; regulatory feasibility; impact evaluation) to de-risk investment. These expectations can be supported through increased representation of regulatory, governance and implementation expertise on funding panels, and would compel researchers and institutions to take translational planning more seriously.

**Create institutional units for end-to-end predictive AI translation and stewardship**

- Academic institutions (and academic-health system partnerships) should recognise the opportunity to support end-to-end predictive AI research and translation. The creation of formal units at institutional or regional level, or expansion of existing technology transfer departments, could bridge existing expertise across the device lifecycle, from research and stakeholder involvement through regulatory certification to commercialisation. These units should embed structured local input from frontline clinicians and health networks (eg, primary care networks and provider collaboratives), aligned to the priorities and governance structures of local health authorities. Initial investment could be recouped by allowing research teams to cost access to the unit within grant applications, improving fundability by evidencing a credible translational pathway, with ongoing postmarket surveillance and model refinement supported through recurring licensing or service agreements.

**Leverage national infrastructure to transform real-world impact**

- Thoughtfully designed, stakeholder-informed predictive AI tools targeting malleable, prioritised clinical problems can lead to benefits that can be felt by patients, health systems and society. Investing in national predictive AI infrastructure as a health system capability to maintain regulatory certification, conduct postmarket activities and support implementation and evaluation at a national scale will bring predictive AI into the realm of patient and population impact. This shifts predictive AI from a predominantly research-based endeavour to a sustained health system function. National institutions must recognise this opportunity to improve national-level health and wealth, and support access to platforms that enable uptake, maintenance and impact evaluation at scale, while allowing flexibility for heterogeneity in local governance structures and population differences.

AI, artificial intelligence.

We acknowledge that empirical evidence on the effectiveness and cost-effectiveness of specific institutional or national models for predictive AI translation is limited and emerging. Implementation and impact should therefore be assessed formally through explicit evaluation plans. These should capture equity, safety, cost and sustainability outcomes, so that health systems can iteratively refine what works. While these recommendations are UK focused, they are likely to generalise or be adaptable to international contexts.

## Conclusion

Predictive AI tools have demonstrable potential to support earlier detection and prevention, yet this potential will not be realised without the infrastructure to enable their safe and sustainable use in practice. While the scientific foundations for predictive AI are increasingly strong, methodological progress alone is insufficient. Coordinated action from funders, institutions and national bodies is needed not only to raise standards for individual studies but to build and sustain the translational pathways that allow promising tools to become living clinical technologies. If translational planning is embedded early, transdisciplinary expertise can be accessed through dedicated units, and national infrastructure is used to support deployment and postmarket oversight at scale, predictive AI research can move beyond publication to deliver genuine patient and population benefit.
